# Supplementary figures and images for: Cell proliferation controls body size growth, tentacle morphogenesis, and regeneration in hydrozoan jellyfish Cladonema pacificum
Source: PeerJ. 2019 Aug 26;7:e7579. doi: 10.7717/peerj.7579 (PMC6714968; doi:10.7717/peerj.7579)

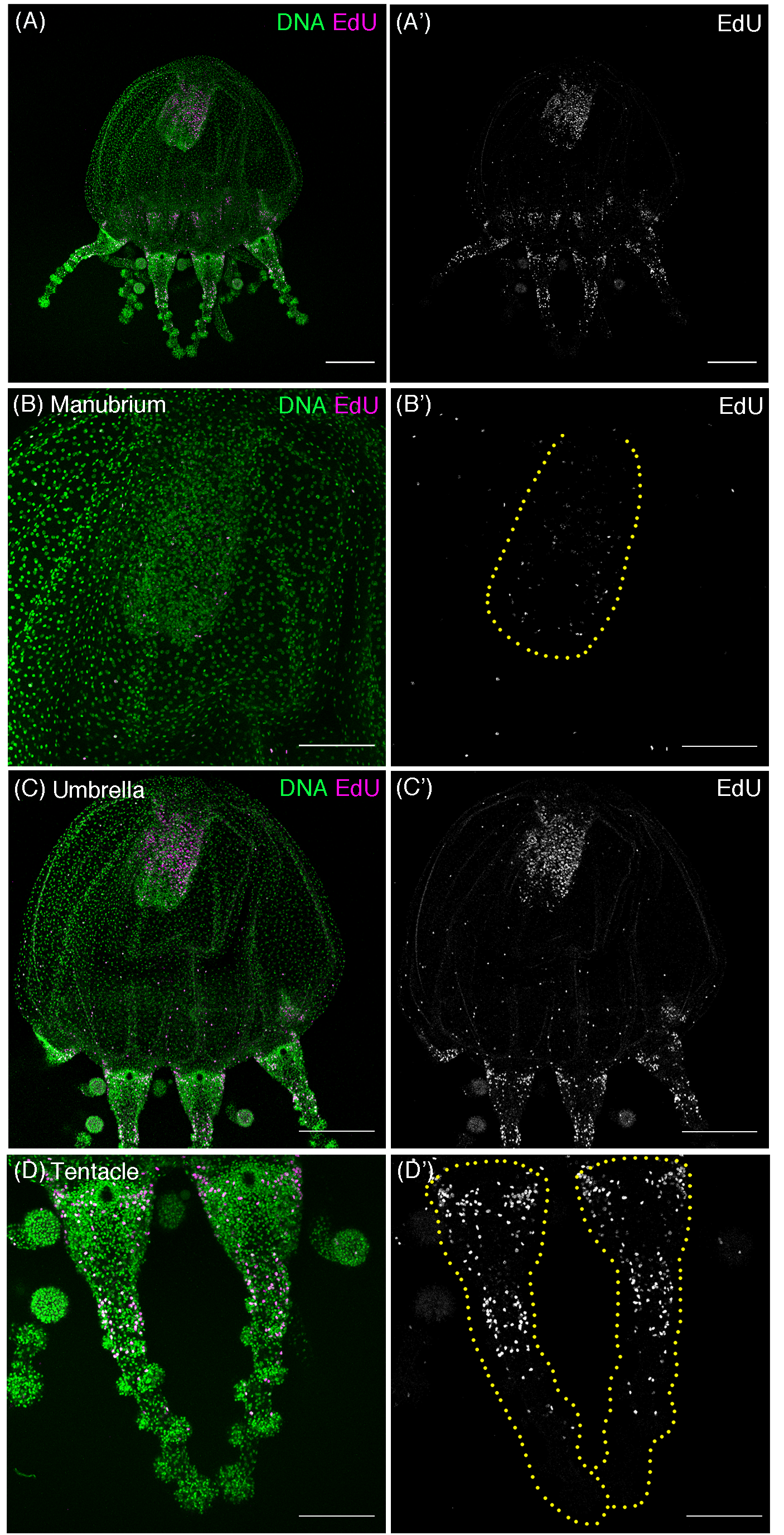

Supplement: Supplemental Information 1 — (A–D) Distribution of S-phase cells in the Cladonema pacificum medusa (1 day old) shown by EdU staining with short incubation time (150 μM, 1 h incubation). (A) Distribution of S-phase cells (EdU+) a whole medusa body. (B) Distribution of S-phase cells (EdU+) in a medusa manubrium. (C) Distribution of S-phase cells (EdU+) in a medusa umbrella. (D) Distribution of S-phase cells (EdU+) in medusa tentacles. Scale bar: (A, C) 200 μm, (B, D) 100 μm. [file peerj-07-7579-s001.png]

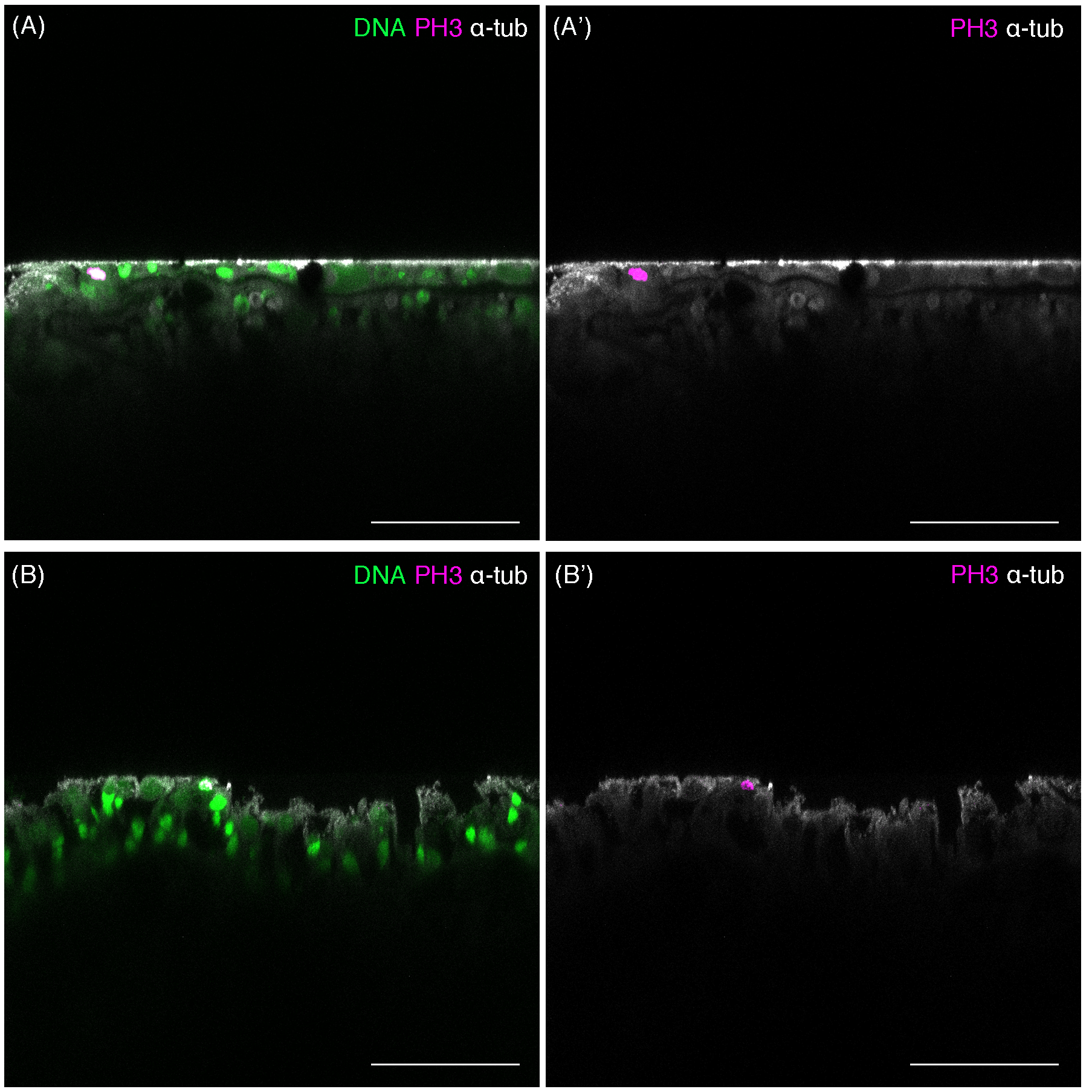

Supplement: Supplemental Information 2 — (A, B) Cross sections of medusa tentacle bulbs. (A) Mitotic cells (PH3+) located in ectoderm in medusa tentacle bulbs (70 days old). (B) Mitotic cells (PH3+) located in ectoderm in medusa tentacle bulbs (30 days old). Scale bars: (A–B) 50 μm. [file peerj-07-7579-s002.png]

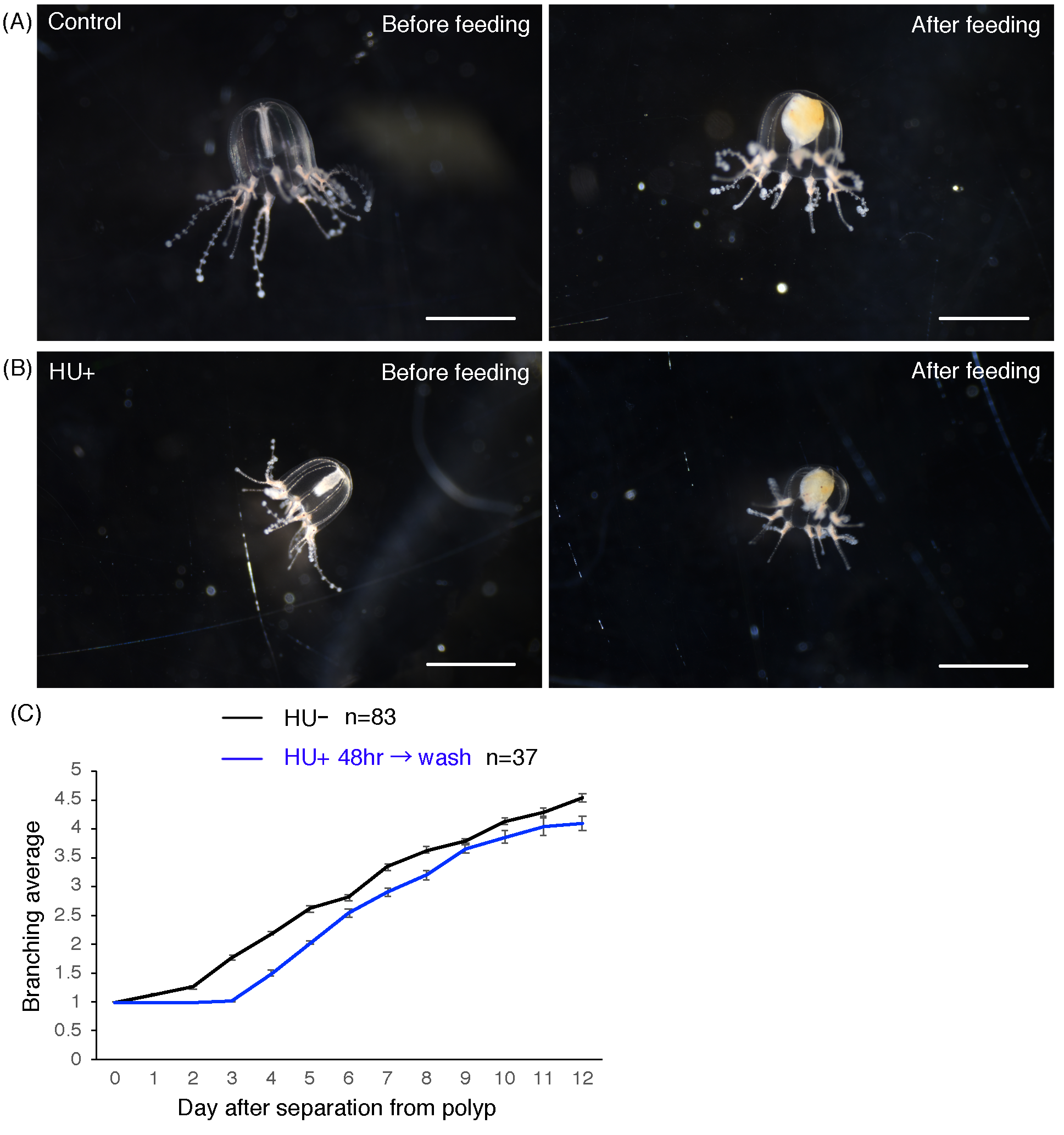

Supplement: Supplemental Information 3 — (A) Cladonema pacificum medusa (2 days old) before feeding (left image) and Cladonema pacificum medusa (2 days old) after feeding (right image). (B) Cladonema pacificum medusa (2 days old) with 48 h HU treatment before feeding (left image) and Cladonema pacificum medusa (2 days old) with 48 h HU treatment after feeding (right image). (C) Quantification of the number of tentacle branching in control and HU-treated medusa, with HU washed off, after 48 h treatment. Error bar: SD. Scale bars: (A, B) one mm. [file peerj-07-7579-s003.png]
